# The Effect of Social Norm-based Intervention with Observable Behaviour on Physical Activity among Adolescents: A Randomized Controlled Trial

**DOI:** 10.1186/s13102-020-00202-y

**Published:** 2020-08-31

**Authors:** Jia Jia LEE, Nivedita Vikas NADKARNI, Irene TEO, Semra OZDEMIR

**Affiliations:** 1 Health Services and Systems Research, Duke-NUS Medical School, 8 College Road, Singapore, 169857 Singapore; 2 Centre for Quantitative Medicine, Duke-NUS Medical School, 8 College Road, Singapore, 169857 Singapore

**Keywords:** Social norms, behaviour observability, physical activity, adolescent

## Abstract

**Background:**

The rising prevalence of childhood obesity in developing and developed countries poses a major public health challenge to policy makers and an effective strategy to promote physical activity among adolescents is warranted. This study aimed to evaluate the effectiveness of providing descriptive norms messages with personal identification in promoting physical activity among adolescents by measuring step counts via a randomized controlled trial (NCT03081013).

**Methods:**

A total of 311 participants aged 13–16 were randomized into two study arms (Onymous and Anonymous Arms). Each arm consisted of 13 groups of 12 participants. During the trial, participants received weekly short message service (SMS) about their past week’s physical activity performance. Participants in the Anonymous Arm received information about step counts of group members ranked from highest to lowest. Participants in the Onymous Arm received the same information with the group members’ full names. Participants’ quality of life, depression, physical activity social support, self-efficacy and enjoyment before and after the intervention were also evaluated. This study adheres to the CONSORT guidelines.

**Results:**

The number of steps was not higher when descriptive norm message was onymous compared to when it was anonymous. Scores in quality of life, depression, social support, self-efficacy, and enjoyment of physical activity were not significantly different between both arms (*p* > 0.05).

**Conclusions:**

Our findings indicated that the effect of providing descriptive norms messages containing personal identification on physical activity promotion was not evident in the main analysis. Future studies may consider using a more relevant reference group to use social norms as a tool to increase physical activity among adolescents.

**Trial registration:**

ClinicalTrials.gov - NCT03081013. Registered 15 Mar 2017-Retrospectively registered, https://clinicaltrials.gov/ct2/show/NCT03081013

## Background

Childhood obesity is one of the major challenges in developed and developing countries where the prevalence of obesity amongst secondary school children has increased in the last few decades [1, 2]. Childhood obesity is a major concern for policy makers because overweight and obese children are likely to remain obese into adulthood [3]. They are also more likely to develop non-communicable diseases at a younger age and have a shorter life expectancy. One of the main reasons behind the growing childhood obesity epidemic is low levels of physical activity. A systematic review showed a high prevalence of insufficient physical activity among adolescents needed for the maintenance and development of cardiovascular health and cardiopulmonary fitness [4]. Previous literature showed evidence of a steep decline in physical activity among boys after primary school years, and low levels of activity among girls throughout primary and high school years, with adolescent years experiencing the largest declines [5, 6]. Given that adolescence is a crucial phase to develop and establish healthy lifestyle behaviours, it is important to explore potential interventions that focuses on increasing physical activity during adolescent years [7].

A strategy that is particularly promising among adolescents is social norm messages [8]. This is based on the notion that individuals use peer norms as a standard to compare their own behaviours. Adolescents care about how they perform against their peers or whether their behaviour is approved by others [9], as childhood (including adolescence) is a formative period when friends are primary points of reference in deciding which behaviours and values are desirable and which are not [10]. In fact, in studies conducted with adolescents, peer influence was found to be a significant correlate of physical activity [11].

Descriptive social norm is the perceptions about the prevalence of a specific behaviour. According to Social Cognitive Theory, perceptions of others’ behaviour lead to behaviour change in an individual [12]. This has been used to change several unhealthy behaviours, such as alcohol and drug use, gambling and recycling [13, 14]. Recent studies have used descriptive norms where study participants were presented with physical activity information of others, and found significant association between receiving descriptive norms and self-reported physical activity, intention to be active or attitude toward physical activity [15–17]. However, none of these studies used an objective assessment of physical activity (e.g., steps directly extracted from pedometers) to measure the study outcome.

Previous studies also showed that descriptive norms feedback is helpful in promoting the intended behaviour for individuals who are worse than the norm. However, this may lead to decline in intended behaviour for individuals who are already better than the norm [18, 19]. Disclosing descriptive norm information with personal identification may overcome this problem. It was reported that individuals are more likely to adhere to norms when their behaviour is observable by other individuals [20, 21]. This could be explained by their desire to fulfil others’ expectations and their concern about how adherence or non-adherence to social norms may affect their reputation. As such, individuals who know that their information will be shared with others may be more motivated to achieve a goal visible to others, regardless of their state of activity.

In this study, we tested whether providing descriptive norm messages with personal identification (Onymous Arm) increases physical activity among adolescents compared to providing descriptive norm messages without personal identification (Anonymous Arm). Adolescents aged 13 to 16 were randomized into one of two arms, and each arm consisted of 13 groups of 12 participants. Participants in the Anonymous Arm received information on the step counts of their group members ranked from highest to lowest, while participants in the Onymous Arm received the same information plus the full names of their group members next to the respective step counts. We hypothesized that the average number of steps taken by adolescents will be higher when descriptive norm message is onymous compared to when it is anonymous. We also investigated the trajectories of physical activity. We hypothesized that, among those in Onymous Arm, girls and those who know other participants in their group will be more likely to be in a trajectory with increasing step counts because perceived peer pressure tends to be stronger among females than among males [22, 23] and among those who know each other compared to those who do not [24]. The secondary aim of this study was to investigate the quality of life, depression, social support, self-efficacy and physical activity enjoyment of participants in both arms before and after the trial.

## Methods

### Recruitment and Eligibility

Between December 2016 to December 2017, 342 participants were recruited on a rolling basis. Recruitment was achieved via a combination of different recruitment strategies such as newspaper advertisement and word-of-mouth. Individuals who were interested were asked to contact the study coordinator for their eligibility. To be eligible for the study, participants must be: (1) Singaporean or Singapore Permanent Resident; (2) between 13 and 16 years old; (3) willing to wear a pedometer for 16 weeks; and (4) English-speaking, which is the dominant language spoken by this age group in Singapore [25–28]. Participants were also screened for exercise-related risks using Physical Activity Readiness Questionnaire (PAR-Q). A doctor’s approval was required if the participant answered “YES” to any of the items in PAR-Q.

Eligible participants were invited to attend a briefing session by the study team at Duke-NUS Medical School Singapore. Informed consent was obtained according to the National University of Singapore-Institutional Review Board (NUS-IRB) protocol, both from the participant and one of the parents or guardians. Consenting participants were then issued a pedometer wristband and asked to complete the baseline assessment that included wearing the pedometer for 2 weeks and answering the baseline survey. The trial lasted 16 weeks and participants were compensated for SGD 50 (~USD 36.23) upon completion of end-of-study survey.

### Sample Size Calculation

The sample size calculation was powered to detect a mean difference of at least 0.25 standard deviations in the average number of steps taken by adolescents between the two study arms, accounting for the correlation between observations at the baseline and at 6 months. Based on 5% level of significance and a power of 80%, and by accounting for a potential 20% attrition, the target sample size was determined to be 312 (156 in each arm).

### Randomization and Intervention Design

**Fig. 1:**
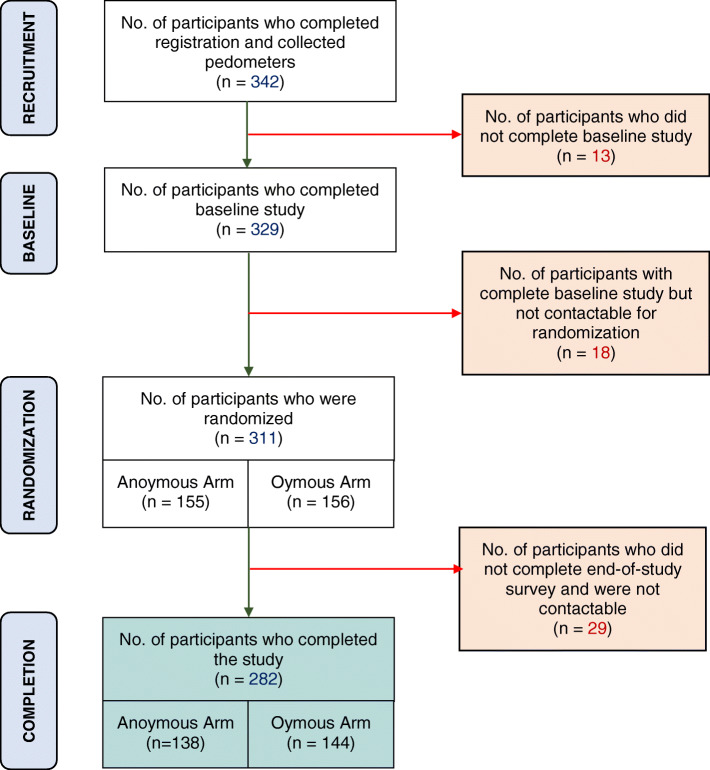
Subject Recruitment

Randomization was performed after baseline assessment was completed. Of the 342 participants who registered for the study, 13 participants did not complete the baseline assessment and were excluded from the study. Eighteen of those who completed the baseline assessment were not contactable and were not included in the study. Participants were randomized based on gender and baseline step counts via block randomization. The randomization algorithm was pre-programmed by a statistician. The allocated arm was sealed in an envelope. A total of 311 participants who completed the baseline assessment (31 dropped out) were randomized to either Anonymous (*n* = 155) or Onymous (*n* = 156) Arms. The participants were informed of their allocated arms after they completed the baseline assessment. Since the aim of the intervention was to provide descriptive norm information about the participants, we could only start the intervention only when all 311 participants were recruited. However, since recruiting 311 participants could have taken us several months and we did not want participants who were recruited earlier in the study to lose interest, we decided to roll out the intervention in batches. A batch included one Anonymous Arm and one Onymous Arm, consisting of 12 participants in each arm. This method resulted in 13 batches in total. We chose 12 participants since we found this number to be large enough to create competition among group members, but small enough that participants can start the trial before they lose interest in the study. Figure 1 shows the recruitment and randomization process.

During the 16-week study period, both study arms received weekly notification of their physical activity performance from the past week via automated short message service (SMS). Participants were ranked according to the total step count accumulated in the past week within their group. For participants in the Anonymous Arm, the weekly information included step count ranked from the highest to lowest within all participants in the same group. Participants in the Onymous Arm received the same information plus the (real) full names of the participants next to the step count. To discourage lower levels of physical activity, those with a step count of zero were not included in the list for both arms.

Participants were asked to complete a survey at both baseline and end of the study. The baseline survey captured socio-demographic information and self-reported physical activity. Paediatric Quality of Life (PedsQL) scale, Asian Adolescent Depression Scale (AADS), Social Support and Exercise scale, Physical Activity Self-Efficacy (PASES) scale, and Physical Activity Enjoyment Scale (PACES-8) were administered in both surveys. The end-of-study survey also included questions about participants’ experience with the study and whether they knew participants in their group and in the study. The survey instruments developed for this study are provided as Additional File 1.

### Measures

#### Steps (primary outcome)

Steps were measured by a Fitbit Flex™ wireless pedometer. Fitbit devices have been validated in measuring step counts among healthy individuals [29]. The step counts recorded by the device can be easily visualized and monitored with a Fitbit account by synchronizing the data to an installed mobile phone or computer application. Average weekly number of steps was used for the analysis.

#### Pediatric Quality of Life (PedsQL) Inventory Score

Quality of life (QoL) was assessed using PedsQL™ 4.0 Generic Core Scales, a 23-item scale that was developed to evaluate QoL in teenagers, and has been widely used in other pediatric QoL studies [30]. The scores were transformed on a scale of 0 to 100. Higher score indicates better QoL [31]. License was obtained to use this scale for this study.

#### Asian Adolescent Depression Scale (AADS)

Depressive symptoms were measured using AADS, a 20-item instrument that was developed in Singapore to assess depression among adolescents [32], and has been successfully used in other studies [33]. The total score is the sum of the 20 items. Possible score for this instrument ranges between 20 to 100. A higher score indicates higher level of depressive symptoms, a total score that exceeds 80 is an indicator of depression [33].

#### Social Support and Exercise (SSE) Survey

Exercise-related support from family and friends was estimated using the Social Support and Exercise Survey [34]. This 13-item instrument evaluates the behaviour and attitude of family and friends toward their participation in exercise in the past 6 months with a 5-point Likert scale. Eleven of the 13 items measure supportive behaviour and attitude toward exercise, while two items measure negative social support associated with exercise. Reverse scoring was performed for the two items, measuring negative social support. A total score was generated by summing scores from both family and friends. Possible score for this scale ranges between 26 to 130. A higher positive support score reflects having received frequent positive support.

#### Physical Activity Self-Efficacy (PASES) Scale

This instrument contains 17 items that assess children’s self-efficacy in overcoming barriers to physical activity [35]. It is a dichotomous scale where “Yes” is coded as “1”, while “No” is coded as “0”. The total score ranges from 0 to 17. Higher score indicates a higher level of self-efficacy associated with physical activity.

#### Physical Activity Enjoyment Scale (PACES-8)

Enjoyment of physical activity was assessed using a set of 8 questions derived from an original 18-item scale to measure enjoyment [36]. Participants were asked to rate their level of exercise-related enjoyment on a 7-point Likert scale. Possible scores for PACES-8 range from 7 to 57, with higher values reflecting greater enjoyment of physical activity.

#### User experience

Participants were asked to report their satisfaction with the intervention and level of participation. We also asked if they know anyone in the study or in their group, and their feelings when they ranked either the top or bottom five.

### Statistical analysis

Analyses were done on an intention-to-treat basis for 311 adolescents who were randomized into one of the arms. The missing data (i.e., zero steps) for the number of steps were imputed based on 30 multiple imputed datasets by employing predictive mean matching [37]. Main analysis of primary outcome was conducted by comparing steps from the 155 participants in Anonymous Arm and 156 participants in the Onymous Arm. Secondary outcomes were analysed based on the completed surveys by the 138 participants in the Anonymous Arm and 144 participants from the Onymous Arm. For the secondary outcomes, scores were not computed if > 50% of the items in the scale were missing [31]. The means of the completed items were imputed only if ≥50% items were completed.

We used a multilevel mixed-effects model to compare the differences between the two arms with random effects for groups and individual effects. In the mixed-effects model, we included the number of steps for the 16 time points, comparing each week in the trial to the baseline.

Group-based trajectory modelling was used to investigate the step trajectories over the trial period [38]. Following the methods from Nagin and Odgers (2010) [38] we identified 4 group trajectories based on the following considerations: 1) obtaining a minimal increase in the Bayes Information Criterion for an additional trajectory group, 2) obtaining for each trajectory group a close correspondence between the estimated probability of group membership and the proportion assigned to that group based on the posterior probability of group membership, 3) ensuring that the average of the posterior probabilities of group membership for individuals assigned to each group exceeds a minimum threshold of 0.7, and 4) ensuring that the odds of correct classification based on the posterior probabilities of group membership exceed a minimum threshold of 5. The functional form of the trajectory for each group was based on the significance of the polynomial terms by iteratively dropping non-significant terms. Dummy variables on gender (female = 1, otherwise = 0) and knowing other participants in the group (know someone = 1, otherwise = 0) were created. They were then interacted with the Onymous Arm dummy variable (Onymous arm = 1, Anonymous arm = 0) and were used as predictors of trajectory group membership. All statistical analysis was performed using Stata 15 [39].

## Results

Table 1 presents baseline characteristics of the two study arms. The average age was 14.6 ± 1.0 years and most participants (42.9%; 95% confidence interval (CI) = 37.3–48.6%) were in their 4th year of secondary school. Consistent with Singapore’s population demographics, most participants were Chinese (89.4%; CI = 85.4–92.6%). There were more females (67.9%; CI = 62.3–73.0%) than males in the study. The median time spent outdoors was 2.5 and 3.5 h per day on weekdays and weekends, respectively. About 45.3% (CI = 39.7–51.1%) and 36.9% (CI = 31–42%) reported exercising more than 1 h on weekdays and weekends, respectively. There were no statistically significant differences between baseline characteristics of the two arms. (*p* > 0.05 for all).

**Table 1:** Baseline Demographics of Study Participants

Characteristics	Total (***n*** = 311)	Anonymous arm (***n*** = 155)	Onymous arm (***n*** = 156)	***p***-value
***Age, Mean*** ± ***SD***	14.6 ± 1.0	14.7 ± 1.0	14.6 ± 1.0	0.799
***Gender, N (%)***				
Female	211 (67.9)	108 (69.7)	103 (66.0)	0.491
***Ethnicity, N (%)***				
Chinese	278 (89.4)	136 (87.7)	142 (91.0)	0.767
Malay/Indian/Others	33 (10.6)	19 (12.3)	14 (9.0)	
***Grade, N (%)***				
Secondary 1	18 (5.8)	10 (6.5)	8 (5.1)	0.493
Secondary 2	61 (19.7)	27 (17.5)	34 (21.8)	
Secondary 3	98 (31.6)	54 (35.1)	44 (28.2)	
Secondary 4	133 (42.9)	63 (40.9)	70 (44.9)	
***Hours spent outdoors (Median, IQR)***				
School day	2.5 (2.5–5.5)	2.5 (1.5–4.5)	3.5 (2.5–5.5)	0.212
Weekend	3.5 (1.5–5.5)	3.5 (1.5–5.5)	3.5 (1.5–7.5)	0.237
***Time spent on sports or exercise while staying outdoors (hours), N (%)***				
*School day*				
None	9 (2.89)	4 (2.6)	5 (3.2)	0.802
Less than 1 h	161 (51.8)	83 (53.6)	78 (50.0)	
More than 1 h	141 (45.3)	68 (43.9)	73 (46.8)	
*Weekend*				
None	38 (12.4)	15 (9.9)	23 (14.9)	0.369
Less than 1 h	155 (50.7)	81 (53.3)	74 (48.1)	
More than 1 h	113 (36.9)	56 (36.8)	57 (37.0)	

Data are presented as mean ± SD, median (IQR) or N (%)

Participants’ total weekly steps and *p*-values for each trial week from the multi-level mixed-effects model are presented in Table 2. In this model, the intervention arms were regarded as a fixed effect, while groups and individuals were regarded as random effects, with individuals nested in the groups. The null hypothesis was not rejected since the number of steps taken by adolescents was not higher when descriptive norm information was onymous compared to when it was anonymous (*p* > 0.05 for all time points).

**Table 2:** Participants’ Total Weekly Steps During the Trial

Time points	Anonymous arm	Onymous arm	Difference between two arms^**a**^	***p***-value^**b**^
***Baseline***	59,926.6 ± 650.7	57,642.4 ± 565.2		
***Week 1***	50,974.9 ± 757.5	51,623.4 ± 656.3	2932.7 ± 2643.3	0.267
***Week 2***	51,558.5 ± 737.9	52,253.6 ± 690.0	2979.3 ± 2734.2	0.276
***Week 3***	52,775.5 ± 740.9	50,290.0 ± 706.7	−201.3 ± 3191.5	0.950
***Week 4***	51,480.4 ± 781.0	53,210.1 ± 765.8	4013.9 ± 3504.4	0.252
***Week 5***	54,376.1 ± 803.8	52,743.6 ± 728.9	651.8 ± 2756.0	0.813
***Week 6***	52,498.8 ± 882.3	53,618.7 ± 766.2	3404.2 ± 3075.9	0.268
***Week 7***	53,541.0 ± 827.2	52,650.1 ± 840.4	1393.4 ± 2842.7	0.624
***Week 8***	52,123.3 ± 840.1	48,994.7 ± 759.0	− 844.4 ± 3186.9	0.791
***Week 9***	53,163.4 ± 889.3	50,114.6 ± 788.3	− 764.5 ± 3152.3	0.808
***Week 10***	51,895.7 ± 929.7	51,497.0 ± 794.7	1885.5 ± 3211.0	0.557
***Week 11***	53,008.3 ± 858.4	52,479.6 ± 881.6	1755.5 ± 3496.3	0.616
***Week 12***	52,500.2 ± 794.5	52,144.8 ± 945.9	1928.8 ± 3330.3	0.562
***Week 13***	53,063.0 ± 891.8	52,385.4 ± 847.6	1606.7 ± 3394.0	0.636
***Week 14***	52,685.7 ± 908.4	51,689.4 ± 864.3	1288.0 ± 2953.9	0.663
***Week 15***	54,081.7 ± 810.1	52,269.8 ± 859.3	472.4 ± 3171.0	0.882
***Week 16***	55,427.0 ± 821.2	52,332.4 ± 965.4	−810.4 ± 3376.9	0.810

^a^Difference between two arms refers to the difference between the two study arms after taking the difference between the baseline step count and the total weekly step count of a particular week

^b^*P* value for the difference between the two arms from mixed-effects model repeated measures; alpha level *p* < 0.05

Table 3 shows the secondary outcomes of both study arms. Compared to participants in the Anonymous Arm, participants in the Onymous Arm reported slightly higher QoL (PedsQL) and lower depression (AADS) scores. However, they reported lower social support for exercise (SSE), lower physical activity self-efficacy (PASES) and lower physical activity enjoyment (PACES) scores compared to Anonymous Arm participants. None of the differences were statistically significant (p > 0.05 for all), except for physical activity self-efficacy score which was significant at the 10% level (*p* = 0.075).

**Table 3:** Comparisons of Secondary Outcomes

	Anonymous arm		Onymous arm		Difference between arms ^a^	***p***-value^**b**^
	Pre-intervention	Post-intervention	Pre-intervention	Post-intervention		
***Total PedsQL score***	78.1 ± 10.6	75.8 ± 12.4	77.4 ± 11.4	76.6 ± 11.7	1.3	0.283
***Total AADS score***	43.7 ± 14.4	47.1 ± 16.4	44.4 ± 14.5	44.7 ± 16.2	− 2.7	0.169
***Total SSE score***	68.7 ± 13.4	67.5 ± 14.7	69.3 ± 14.4	66.2 ± 14.0	−1.8	0.192
***Total PASES score***	12.5 ± 2.7	12.4 ± 3.0	13.0 ± 2.7	12.5 ± 2.9	−0.5	0.075
***Total PACES score***	42.8 ± 8.4	42.9 ± 7.8	42.6 ± 8.4	42.0 ± 7.4	−1.0	0.237

Data are presented as mean ± SD

^a^Difference between two arms refers to the difference between Onymous and Anonymous arms after taking the difference between the pre-intervention and post-intervention outcomes

^b^The *p*-values for the difference between the two arms from the mixed-effects model; alpha level *p* < 0.05

Table 4 presents the user experience reported by participants at the end of study. Majority of the participants were somewhat satisfied with this intervention, with no significant difference between the two study arms (Anonymous: 63.8%, CI = 55.2–71.8%; Onymous: 63.2%, CI = 54.8–71.1%, *p* = 0.774). About two-thirds of the participants reported wearing the pedometer nearly every day (Anonymous: 66.7%, CI = 58.1–74.5%; Onymous: 63.9%, CI = 55.5–71.7%, *p* = 0.542). Most participants reported that they checked the weekly ranking SMS sent to them every week (Anonymous: 67.4%, CI = 58.9–75.1%; Onymous: 64.6%, CI = 56.2–72.4%, *p* = 0.591). When ranked in the top five, most participants stated that they felt accomplished (Anonymous: 79.0%, CI = 70.9–85.3%; Onymous: 76.5%, CI = 68.5–83.0%, *p* = 0.890) or felt good (Anonymous: 84.7%, CI = 77.2–90.0%; Onymous: 74.2%, CI = 66.1–81.0%, *p* = 0.111). When ranked in the bottom five, the most commonly reported response was ‘not a big deal’ (Anonymous: 51.6%, CI = 42.8–60.3%; Onymous: 51.4%, CI = 43.1–59.7%, *p* = 0.800) or ‘did not care’ (Anonymous: 32.3%, CI = 24.6–41.0%; Onymous: 31.2%, CI = 24.0–39.4%, *p* = 0.646). Of note, there was a significant difference between two arms in knowing someone in the same group. There were significantly more participants in the Onymous arm who reported that they knew someone in the same study group (Anonymous: 21.7%, CI: 15.6–29.4%; Onymous: 50.7%, CI: 42.5–58.8%).

**Table 4:** User Experience (End-of-study Survey)

	Total (***n*** = 282)	Anonymous arm (***n*** = 138)	Onymous arm (***n*** = 144)	***p***-value
***Satisfaction with intervention,*** **N (%)**				
Very satisfied	91 (32.3)	44 (31.9)	47 (32.6)	0.774
Somewhat satisfied	179 (63.5)	88 (63.8)	91 (63.2)	
Somewhat dissatisfied	11 (3.9)	6 (4.4)	5 (3.5)	
Very dissatisfied	1 (0.4)	0 (0.0)	1 (0.7)	
***Frequency of wearing the pedometer,*** **N (%)**				
Nearly every day	184 (65.3)	92 (66.7)	92 (63.9)	0.542
More than half the days	72 (25.5)	32 (23.2)	40 (27.8)	
Less than half the days	18 (6.4)	11 (8.0)	7 (4.9)	
Rarely wore it	8 (2.8)	3 (2.2)	5 (3.5)	
***Frequency of checking weekly ranking SMS,*** **N (%)**				
Every week	186 (66.0)	93 (67.4)	93 (64.6)	0.591
Almost every week	53 (18.8)	24 (17.4)	29 (20.1)	
Some weeks	29 (10.3)	12 (8.7)	17 (11.8)	
Only a few weeks	10 (3.6)	7 (5.1)	3 (2.1)	
Never	4 (1.4)	2 (1.5)	2 (1.4)	
***Knowing someone in the same group,*** **N (%)**	103 (36.5)	30 (21.7)	73 (50.7)	0.000
***Knowing someone in the study but not in the same group,*** **N (%)**	236 (83.7)	112 (81.2)	124 (86.1)	0.261
***No. of participants who had ever been ranked in top five,*** **N (%)**	256 (90.8)	124 (89.9)	132 (91.7)	0.599
***How did participants feel on weeks when they were ranked in top five,*** **N (%)**				
I felt accomplished	199 (77.7)	98 (79.0)	101 (76.5)	0.890
I felt good	203 (79.3)	105 (84.7)	98 (74.2)	0.111
I felt proud	156 (60.9)	85 (68.6)	71 (53.8)	0.053
I thought it was no big deal	105 (41.0)	48 (38.7)	57 (43.2)	0.133
I did not care	48 (18.8)	23 (18.6)	25 (18.9)	0.336
***No. of participants who had ever been ranked lower than top five,*** **N (%)**	235 (92.2)	109 (88.6)	126 (95.5)	0.042
***How did participants feel on weeks when they were ranked lower than top five,*** **N (%)**				
I felt I failed	24 (9.2)	11 (8.9)	13 (9.4)	0.826
I felt bad	52 (19.9)	26 (21.0)	26 (18.8)	0.908
I felt disappointed	60 (22.9)	34 (27.4)	26 (18.8)	0.243
I thought it was no big deal	135 (51.5)	64 (51.6)	71 (51.5)	0.800
I did not care	83 (31.7)	40 (32.3)	43 (31.2)	0.646

Data are presented as n (%)

**Fig. 2:**
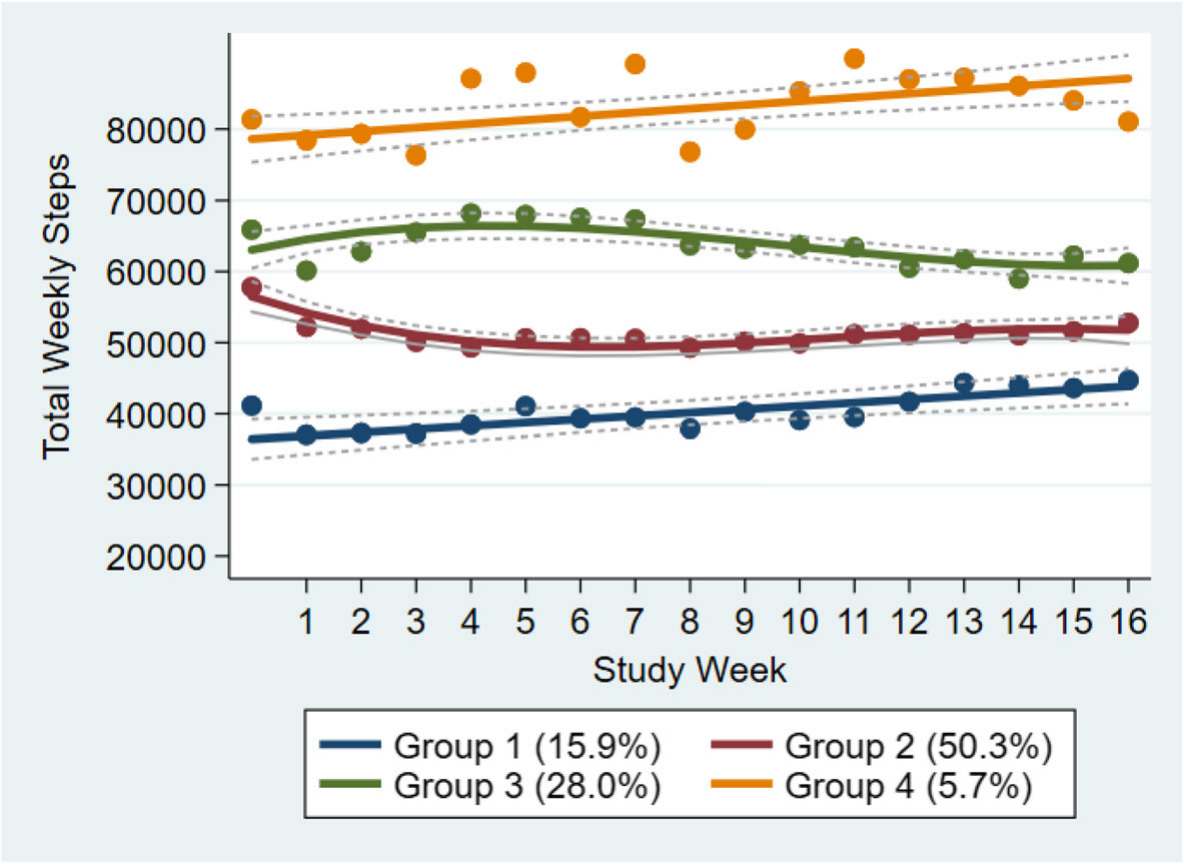
Total Weekly Step Trajectories (n = 311)

Figure 2 shows the trajectories of weekly steps over the trial course for the pooled data from both arms. Results show that a 4-group trajectory class was the best fit to the data. Group 1 consisting of 15.9% of the participants and Group 4 consisting of 5.7% of the participants made an effort to increase their physical activity. However, participants in Group 1 started at a much lower step counts at baseline and maintained the lower step counts when compared with other groups. In contrast, Group 4 logged in the highest step counts in the sample. Group 2 (50.3% of the participants) and 3 (28% of the participants) had similar step counts at baseline. Group 2 reduced their steps in the first 4 weeks and remained stable for the rest of the trial. On the other hand, Group 3 increased their steps in the first 4 weeks but decreased slightly in the remaining weeks. Table 5 shows the multinomial logit model estimates for the predictors of trajectory group membership. Compared with Group 1 (reference group), Onymous Arm female participants were more likely to be in Group 3 (beta: 2.418, SE = 0.706; *p* = 0.001) and in Group 4 (beta: 2.650, SE = 0.942; *p* = 0.005). Knowing someone in the same group in Onymous Arm was not a significant predictor (*p* > 0.05 for all).

**Table 5:** Predictors of Trajectory Group Membership – Results from the Multinomial Logit Model

Variable	Coefficient	Standard error	***p***-value
***Group 2***			
Constant	1.195	0.304	0.000
Onymous arm	−0.311	0.463	0.501
Female * Onymous	0.457	0.691	0.509
Know someone * Onymous	0.288	0.526	0.584
***Group 3***			
Constant	0.701	0.319	0.028
Onymous arm	−1.562	0.575	0.007
Female * Onymous	2.418	0.706	0.001
Know someone * Onymous	0.854	0.611	0.162
***Group 4***			
Constant	−1.082	0.447	0.016
Onymous arm	−1.167	0.895	0.193
Female * Onymous	2.650	0.942	0.005
Know someone * Onymous	0.559	0.853	0.512
BIC	−58,297		

* signifies the interaction between variables

## Discussion

Based on our findings, the null hypothesis cannot be rejected. The average number of steps taken by adolescents was not higher when social norm message was onymous compared to when it was anonymous. There were also no significant differences between the two arms in QoL, depression, social support for exercise, physical activity self-efficacy and physical activity enjoyment scores.

The normative reference group used in the study may explain the lack of effectiveness of the descriptive norms used in our study. In this study, participants were recruited on an individual basis and they did not share a proximal social network. Although 36.5% reported that they knew someone in the same group, the person that they knew may not have been in their close social network. It is possible that adolescents in our sample did not care about being compared to other adolescents who were not in their close social network. In fact, majority of the reported reaction to being ranked at the bottom five of a group was that it was not a big deal or that they did not care. Descriptive norms might have been more effective in persuading subjects to adopt target behaviour if the normative reference group was a more relevant social group such as close friends, or peers that they interact regularly with such as classmates. According to social identification theory, not all groups are equal and an individual’s behaviour is influenced by the group to which he or she perceives a sense of belonging [40, 41]. In addition, a norm describing the behaviour of a relevant or salient social group leads people to identify more with and feel more self-efficacious regarding this particular target behaviour [24, 42, 43]. Therefore, it is possible that the effect of norms on increasing self-efficacy in relation to physical activity can only be activated by increasing the saliency or relevance of the normative reference group.

Although the effect of descriptive norms was not evident in the main analysis, further analysis using group-based trajectory analysis revealed that being a female in the Onymous Arm was a significant predictor of trajectory group membership with increased step counts. Females were more sensitive to social norm messaging at the beginning of the trial. This implies that social norms may not be effective for all adolescents, and interventions targeting specific groups may be more successful in promoting physical activity.

One concern is that providing participant full names together with the step count information may lead to negative outcomes for adolescents. In fact, participants in the Onymous Arm where participants were identified with their full names reported slightly worse outcomes in social support for exercise, physical activity self-efficacy, and physical activity enjoyment scores compared to those in the Anonymous Arm. Participants who logged in lower step counts might have questioned their ability to achieve higher step counts and not enjoyed physical activity when they were ranked low in their group, knowing that others had seen this information. However, the differences were statistically significant at the 10% level only for the physical-activity self-efficacy. In addition, participants in both groups did not report significantly different responses on how they felt when they were ranked in the top 5 or bottom 5. These findings imply that providing full names did not lead to negative outcomes, although it did not contribute to a higher step count.

Our study has several limitations. Some participants stopped wearing their pedometers before the study ended, which resulted in incomplete data. However, we conducted intent-to-treat analyses to account for this. Second, it is possible to generate step counts on pedometers without the user walking or running. We tried to minimize this possibility by asking participants to sign a statement of oath to log their steps accurately and honestly. Third, our findings were based on a very specific descriptive norm messaging in one type of setting. Different framing or methods of presenting descriptive norms with more relevant reference group may be more successful in promoting physical activity among adolescents.

To our knowledge, this is the first study that measured the effect of descriptive norms objectively by using step count directly extracted from pedometers. Previous studies that used descriptive norms to promote physical activity have used either self-reported activity levels or intention to exercise as primary outcomes, which could potentially over-reported physician activity as a result of social desirability bias [44]. Although the implementation of descriptive norms are different between our study and other studies, the difference in findings suggests that conclusion based on subjective measures of physical activity may not be an accurate reflection of the actual physical activity as participants might have over-reported experienced recall bias on the amount or intent of physical activity. In addition, this study contributes to the knowledge concerning whether the effect of descriptive norms can be amplified when the targeted behaviour is observable. Future studies should explore different framing of descriptive norms and we recommend using a more relevant reference group to facilitate social norms among adolescents.

## Conclusions

The effect of descriptive norms messages containing personal identification on physical activity promotion was not evident in the main analysis. As adolescence is an important phase for the cultivation of healthy lifestyle, it is important to explore interventions that could motivate adolescents to increase their physical activity. Future study may consider exploring the effect of similar intervention among adolescents using a more relevant reference group.

## Supplementary information

**Additional file 1.** Questions in the baseline and end-of-study survey that were developed by authors.

## Data Availability

The datasets used and/or analysed during the current study are available from the corresponding author on reasonable request.

## Abbreviations

SMS: Short message service
PAR-Q: Physical activity readiness questionnaire
NUS-IRB: National University of Singapore-Institutional Review Board
SGD: Singapore dollar
USD: United States dollar
PedsQL: Paediatric quality of life
AADS: Asian adolescent depression scale
PASES: Physical activity self-efficacy scale
PACES-8: Physical activity enjoyment scale – 8 items
QoL: Quality of life
SSE: Social support and exercise
CI: Confidence interval
SD: Standard deviation
JTC: Jurong town corporation
IQR: Inter-quartile range

## References

[1] Gupta N, Goel K, Shah P, Misra A (2012). Childhood obesity in developing countries: epidemiology, determinants, and prevention. Endocr Rev.

[2] Ng M, Fleming T, Robinson M, Thomson B, Graetz N, Margono C (2014). Global, regional, and national prevalence of overweight and obesity in children and adults during 1980–2013: a systematic analysis for the Global Burden of Disease Study 2013. Lancet.

[3] Health Promotion Board. Obesity. HPB-MOH Clinical Practice Guidelines 1/2016. 2016.

[4] Moraes ACFd, Guerra PH, Menezes PR. The worldwide prevalence of insufficient physical activity in adolescents; a systematic review. Nutr Hosp. 2013;28(3):575–84. 10.3305/nh.2013.28.3.6398 23848074

[5] Chia M (2010). Pedometer-assessed physical activity of Singaporean youths. Prev Med.

[6] Armstrong N, Welsman JR (2006). The physical activity patterns of European youth with reference to methods of assessment. Sports Med.

[7] Sawyer SM, Afifi RA, Bearinger LH, Blakemore S-J, Dick B, Ezeh AC (2012). Adolescence: a foundation for future health. Lancet.

[8] Sharps M, Robinson E (2015). Perceived eating norms and vegetable consumption in children. Int J Behav Nutr Phys Act.

[9] Kirkcaldy BD, Shephard RJ, Siefen RG (2002). The relationship between physical activity and self-image and problem behaviour among adolescents. Soc Psychiatry Psychiatr Epidemiol.

[10] Maturo CC, Cunningham SA (2013). Influence of friends on children’s physical activity: a review. Am J Public Health.

[11] Baker CW, Little TD, Brownell KD (2003). Predicting adolescent eating and activity behaviors: the role of social norms and personal agency. Health Psychol.

[12] Bandura A (1986). National Institute of Mental Health. Social foundations of thought and action: a social cognitive theory.

[13] Donaldson SI, Graham JW, Hansen WB (1994). Testing the generalizability of intervening mechanism theories: Understanding the effects of adolescent drug use prevention interventions. J Behav Med.

[14] Larimer ME, Neighbors C (2003). Normative misperception and the impact of descriptive and injunctive norms on college student gambling. Psychol Addict Behav.

[15] Koeneman MA, Chorus A, Hopman-Rock M, Chinapaw MJ (2017). A novel method to promote physical activity among older adults in residential care: an exploratory field study on implicit social norms. BMC Geriatr.

[16] Priebe CS, Spink KS (2011). When in Rome: Descriptive norms and physical activity. Psychol Sport Exerc.

[17] Van Bavel R, Esposito G, Baranowski T, Duch-Brown N (2017). Tracing how normative messages may influence physical activity intention. J Sport Exerc Psychol.

[18] Frey BS, Meier S (2004). Pro-social behavior in a natural setting. J Econ Behav Organ.

[19] Shang J, Croson R (2009). A field experiment in charitable contribution: The impact of social information on the voluntary provision of public goods. Econ J.

[20] Schram A, Charness G (2015). Inducing social norms in laboratory allocation choices. Manag Sci.

[21] Vesely S, Klöckner CA (2018). How anonymity and norms influence costly support for environmental causes. J Environ Psychol.

[22] Hutchinson DM, Rapee RM (2007). Do friends share similar body image and eating problems? The role of social networks and peer influences in early adolescence. Behav Res Ther.

[23] McCabe MP, Ricciardelli LA (2005). A prospective study of pressures from parents, peers, and the media on extreme weight change behaviors among adolescent boys and girls. Behav Res Ther.

[24] Cho H (2006). Influences of norm proximity and norm types on binge and non-binge drinkers: examining the under-examined aspects of social norms interventions on college campuses. J Subst Abus.

[25] Ramiah K. The pattern of Tamil language use among primary school Tamil pupils in Singapore. 1991.

[26] Pillai AD (2009). Language shift among Singaporean Malayalee families. Lang India.

[27] Cavallaro F, Serwe SK (2010). Language use and language shift among the Malays in Singapore. Appl Linguist Rev.

[28] Saravanan LWV, Hoon JNL (1997). Language shift in the Teochew community in Singapore: A family domain analysis. J Multiling Multicult Dev.

[29] Adam Noah J, Spierer DK, Gu J, Bronner S (2013). Comparison of steps and energy expenditure assessment in adults of Fitbit Tracker and Ultra to the Actical and indirect calorimetry. J Med Eng Technol.

[30] Elalfy MS, Farid MN, Labib JH, RezkAllah HK (2014). Quality of life of Egyptian b-thalassemia major children and adolescents. Egypt J Haematol.

[31] Varni JW. The PedsQL™ Measurement Model for the Pediatric Quality of Life Inventory™. 1998.

[32] Woo BS, Chang WC, Fung DS, Koh JB, Leong JS, Kee CH (2004). Development and validation of a depression scale for Asian adolescents. J Adolesc.

[33] Yi S, Poudel KC, Yasuoka J, Palmer PH, Yi S, Jimba M (2010). Role of risk and protective factors in risky sexual behavior among high school students in Cambodia. BMC Public Health.

[34] Sallis JF, Grossman RM, Pinski RB, Patterson TL, Nader PR (1987). The development of scales to measure social support for diet and exercise behaviors. Prev Med.

[35] Saunders RP, Pate RR, Felton G, Dowda M, Weinrich MC, Ward DS (1997). Development of questionnaires to measure psychosocial influences on children's physical activity. Prev Med.

[36] Raedeke TD (2007). The relationship between enjoyment and affective responses to exercise. J Appl Sport Psychol.

[37] White IR, Royston P, Wood AM (2011). Multiple imputation using chained equations: issues and guidance for practice. Stat Med.

[38] Nagin DS, Odgers CL (2010). Group-based trajectory modeling in clinical research. Annu Rev Clin Psychol.

[39] Cooperation S (2017). Stata 15.

[40] Michael AH, Dominic A. Social identifications: a social psychology of intergroup relations and group processes. Methuen. 1988.

[41] Tajfel H, Turner J (1986). The social identity theory of intergroup behaviour. u: Worchel S. i Austin WG (ur.) Psychology of intergroup relations.

[42] Stok FM, de Vet E, de Ridder DT, de Wit JB (2016). The potential of peer social norms to shape food intake in adolescents and young adults: a systematic review of effects and moderators. Health Psychol Rev.

[43] Stok FM, Verkooijen KT, de Ridder DT, de Wit JB, de Vet E (2014). How norms work: Self-identification, attitude, and self-efficacy mediate the relation between descriptive social norms and vegetable intake. Appl Psychol Health Well-Being.

[44] Sallis JF, Strikmiller PK, Harsha DW, Feldman HA, Ehlinger S, Stone EJ (1996). Validation of interviewer-and self-administered physical activity checklists for fifth grade students. Med Sci Sports Exerc.

