# Supplementary material for: The Effect of Social Norm-based Intervention with Observable Behaviour on Physical Activity among Adolescents: A Randomized Controlled Trial
Source: BMC Sports Sci Med Rehabil. 2020 Aug 31;12:52. doi: 10.1186/s13102-020-00202-y (PMC7457489; doi:10.1186/s13102-020-00202-y)
Supplement: Supplementary file 1 — Additional file 1. Questions in the baseline and end-of-study survey that were developed by authors. [file 13102_2020_202_MOESM1_ESM.pdf]

Additional File 1

Questions in the baseline survey that were developed by authors

Section 1: Socio-demographic questions

Label	Question	Filter
age	How old were you on your last birthday? [Numeric]	

Label	Question	Filter
gender	Are you…? [SC]	
1	Male	
2	Female	

Label	Question	Filter
ethnic	Which ethnic group do you belong to? [SC]	
1	Chinese	
2	Malay	
3	Indian	
4	Other (please specify: _______________________)	

Label	Question	Filter
tenure	What type of housing are you living in? [SC]	
1	HDB/ JTC flat (1-2 room)	
2	HDB/ JTC flat (3 room)	
3	HDB/ JTC flat (4 room)	
4	HDB/ JTC flat (5 room and above/ HUDC/ Executive)	
5	Condominium/ Private flat	
6	Bungalow/ semi-detached/ terrace house	
7	Shophouse	
8	Other (please specify: _______________________)	

Label	Question		Filter
hincome	Please tell me which letter best describes your **household's total income from all sources**, after tax and compulsory deductions? [SC]		
	**Monthly**	**Yearly**	
1	Below $1,000	Below $12,000	
2	$1,000-$1,999	$12,000-$23,999	
3	$2,000-$2,999	$24,000-$35,999	
4	$3,000-$3,999	$36,000-$47,999	
5	$4,000-$4,999	$48,000-$59,999	
6	$5,000-$5,999	$60,000-$71,999	
7	$6,000-$6,999	$72,000-$83,999	
8	$7,000-$9,999	$84,000-$119,999	
9	$10,000 and over	$120,000 and over	
999	Prefer not to say		
777	Don't know		

Label	Question	Filter
chclass	Which grade do you study in? [SC]	
1	Secondary 1	
2	Secondary 2	
3	Secondary 3	
4	Secondary 4	

Label	Question	Filter
odwd	On a typical school day, how many hours do you spend outdoors? [Numeric]	GO TO odwe IF ‘0’

Label	Question	Filter
odwdsport	**WHILE OUTDOORS**, for how long do you play sports or exercise (including school co-curricular activities) [SC]	
1	Not at all	
2	< 1 hour	
3	> 1 hour<br>(please specify number of hours: ______________)	

Label	Question	Filter
odwe	On a typical weekend, how many hours do you spend outdoors in a day? [Numeric]	GO TO allow IF ‘0’

Label	Question	Filter
odwesport	**WHILE OUTDOORS**, for how long do you play sports or exercise (including school co-curricular activities) [SC]	
1	Not at all	
2	< 1 hour	
3	> 1 hour<br>(please specify number of hours: ___________________)	

Questions in the end-of-study survey that were developed by the authors

Section 6: Evaluation questionnaire

Label	Question	Filter
oversat	Overall, how satisfied are you with the FIT-TEEN study? [SC]	
1	Very satisfied	
2	Somewhat satisfied	
3	Somewhat dissatisfied	
4	Very dissatisfied	

Label	Question	Filter
pedfreq	How often did you wear your Fitbit in the past 4 months during the study? [SC]	
1	Nearly every day	
2	More than half the days	
3	Less than half the days	
4	Rarely wore it	
5	Never wore it	

Label	Question	Filter
smscheck	How often did you check the weekly ranking information that was sent to you by SMS during the study?	
1	Every week	
2	Almost every week	
3	Some weeks	
4	Only a few weeks	
5	Never	

Label	Question	Filter
pedacc	How often did you log into your **Fitbit** account page to check your stats in the past 4 months during the study? [SC]	
1	Nearly every day	
2	2 -3 times a week	
3	Once a week	
4	Once a month	
5	Rarely checked it	
6	Never checked it	

Label	Question	Filter
studyacc	How often did you log into your **FIT-TEEN** account page to check your stats in the past 4 months during the study? [SC]	
1	Nearly every day	
2	2 -3 times a week	
3	Once a week	
4	Once a month	
5	Rarely checked it	
6	Never checked it	

Label	Question	Filter
samegr	Do you know anyone in the same FIT-TEEN group as you?	
1	Yes	
2	No	

Label	Question [If yes to samegr]	
nsame	How many participants do you know in your FIT-TEEN group?	
	____________________	

Label	Question	
knowso	Do you know anyone participating in the FIT-TEEN study but not in your group?	
1	Yes	
2	No	

Label	Question [If yes to knowso]	
nso	How many participants do you know in the FIT-TEEN study but not in your group?	
	______________________	

Label	Question	1	2	3	4	5
Feeltop	How did you feel on the weeks when you found out that you were ranked in the top 5?	**Strongly Disagree**	**Disagree**	**No opinion**	**Agree**	**Strongly Agree**
1	I felt accomplished					
2	I felt good					
3	I felt proud					
4	I thought it was no big deal					
5	I did not care					
6	I have never made it to top 5					

Label	Question	1	2	3	4	5
Feelbottom	How did you feel on the weeks when you found out that you were ranked in the bottom 5?	**Strongly Disagree**	**Disagree**	**No opinion**	**Agree**	**Strongly Agree**
1	I felt I failed					
2	I felt bad					
3	I felt disappointed					
4	I thought it was no big deal					
5	I did not care					
6	I was never at the bottom 5					
